# The paradoxical role of stem cells in osteosarcoma: from pathogenesis to therapeutic breakthroughs

**DOI:** 10.3389/fonc.2025.1643491

**Published:** 2025-09-16

**Authors:** Zhengbing Su, Xiang Fang, Hong Duan

**Affiliations:** Department of Orthopedic Surgery, West China Hospital, Sichuan University, Chengdu, China

**Keywords:** osteosarcoma, cancer stem cells, mesenchymal stem cells, regulatory mechanisms, translational barriers

## Abstract

Osteosarcoma (OS), the most prevalent primary malignant bone tumor in adolescents, exhibits a high metastatic potential and resistance to therapy. This characteristic results in a dismal prognosis in advanced cases even following multimodal therapies. This review synthesizes the dual roles of stem cells in OS pathogenesis and therapeutic innovation. Cancer stem cells (CSCs) drive tumor initiation, progression, and chemoresistance through dysregulated molecular pathways that include Wnt/β-catenin, Notch, and Hedgehog signaling, with key markers such as CD133 and CXCR4 contributing to stemness maintenance and metastasis. Concurrently, mesenchymal stem cells (MSCs) paradoxically influence OS progression. Although their tumor-homing capacity enables targeted drug delivery (e.g., IDD-1040-paclitaxel complexes) and immunomodulation, MSC-derived factors like TGF-β can promote cancer-associated fibroblast differentiation and immune evasion. The immunosuppressive tumor microenvironment (TME), characterized by hypoxia-induced HIF-1α activation, metabolic reprogramming, and M2 macrophage polarization, further facilitates CSC resilience and therapy resistance. Emerging strategies—including CSCs-targeted agents (AZD1080, DNMTi/HDACi), CRISPR/Cas9-engineered CD133-directed CAR-T cells, and MSC-mediated delivery of oncolytic viruses—show preclinical promise in overcoming these barriers. However, critical challenges persist: intratumoral CSC heterogeneity limits targeted therapy efficacy; MSC functional plasticity risks tumor promotion via fusion or batch variations; and inefficient cell homing due to pulmonary entrapment reduces therapeutic delivery. Future directions necessitate biomarker-guided combinatorial approaches, optimized MSC administration routes (e.g., intra-arterial injection), and integrated multi-omics profiling to address translational bottlenecks. Resolving these issues will advance personalized stem cell-focused therapies for OS.

## 1 Introduction

Osteosarcoma (OS), the most common primary malignant bone tumor in adolescents and young adults, is characterized by its aggressive nature and propensity for metastasis. Despite significant advancements in multimodal therapies combining surgery, chemotherapy, and radiotherapy, the prognosis for patients with metastatic or chemotherapy-resistant disease remains dismal, with survival rates stagnating below 20% over the past decades (1). This therapeutic plateau underscores the urgent need to unravel the molecular underpinnings of OS pathogenesis and resistance mechanisms, which is increasingly linked to the dynamic interplay between tumor cells and their microenvironment (2).

Currently, there are still many crucial issues in osteosarcoma research that urgently need to be addressed. On the one hand, the heterogeneity of cancer stem cells (CSCs) and the mechanisms underlying the maintenance of their stemness have not been fully elucidated. Although pathways such as Wnt/β-catenin and Notch are known to be involved in regulation, the differences in the dependence of different CSCs sub-populations (such as CD133+ and CXCR4+) on these pathways, as well as their specific roles in chemoresistance and metastasis, remain controversial (3). On the other hand, the dual role of mesenchymal stem cells (MSCs) constitutes the core contradiction in clinical translation. Their tumor-homing ability makes them an ideal drug delivery vehicle, but some studies have confirmed that MSCs can induce the differentiation of cancer-associated fibroblasts (CAFs) through paracrine factors such as TGF-β, which instead promotes tumor progression. The balance mechanism between this “tumor-promoting” and “tumor-suppressing” effect has not been clarified. In addition, the dynamic regulatory network of the tumor microenvironment (TME) still needs to be analyzed. For example, the molecular details of how hypoxia upregulates the stemness of CSCs through HIF-1α, and how the interactions between immune cells (such as M2-type macrophages) and stem cells mediates immune escape are all weak links in current research (4). Among existing treatment strategies, CSCs-targeted drugs have poor efficacy due to heterogeneity. MSC-mediated drug delivery faces problems such as short cell survival time and low targeting efficiency (5). Emerging technologies such as CRISPR/CAR-T are limited by issues like antigen loss and off-target effects. Overcoming these bottlenecks is critical for improving patient prognosis (7). This review systematically synthesizes the latest advances in understanding the pathological mechanisms of OS, emphasizing the dual roles of stem cells in disease progression and therapeutic innovation. By dissecting the molecular crosstalk between CSCs, MSCs, and the TME, we critically evaluate emerging strategies such as CRISPR-engineered Chimeric Antigen Receptor T-Cell (CAR-T cells), epigenetic modulators, and optimized MSC delivery routes. Finally, we propose future directions to overcome translational barriers, advocating for multidisciplinary approaches that bridge preclinical insights with clinical realities to improve outcomes for OS patients (6).

This review systematically synthesizes the latest advancements in understanding the pathological mechanisms of OS, highlighting the dual roles of stem cells in disease progression and therapeutic innovation. By dissecting the molecular interactions among cancer stem cells (CSCs), mesenchymal stem cells (MSCs), and the tumor microenvironment (TME), we critically evaluate emerging strategies such as CRISPR-engineered chimeric antigen receptor T cells (CAR-T cells), epigenetic regulators, and optimized mesenchymal stem cell delivery pathways. Finally, we propose future directions to overcome translational barriers, advocating for a multidisciplinary approach that integrates pre-clinical research findings with clinical practice to improve treatment outcomes for osteosarcoma patients.

## 2 Pathological overview of osteosarcoma

### 2.1 Epidemiology and clinical features

OS is the most common primary malignant bone tumor, which is divided into primary (central or surface) and secondary OS, originating from previous diseases (7). According to differences in biological behavior, primary bone tumors can be further classified into benign lesions and malignant entities, which have significant differences in proliferation characteristics, clinical symptoms, and treatment sensitivity (8). This classification system has important guiding significance for the clinical development of individualized diagnosis and treatment plans (9). Among malignant primary bone tumors, OS has the highest incidence. Its peak incidence coincides with the rapid bone development stage during adolescence, and the patient population is mainly adolescents and young people (10). The typical pathological feature of this tumor is the uncontrolled differentiation of abnormally proliferating mesenchymal cells into osteoblast-like cells, which secrete a large amount of immature bone matrix or osteoid tissue (11).

OS originates from osteogenic MSCs and has a higher incidence in the metaphysis of the distal femur, proximal tibia and proximal humerus (12). In clinical practice, persistent local pain and venous dilation are its main manifestations. When systemic cachexia appears, it will pose a serious threat to the patient’s life.

OS often carries total genomic mutations and rearrangements, including chromosomal translocations (13–15). Chromosome aberrations are observed in thousands of clusters of chromosomal rearrangements in 25% of clinical OS human samples, while in cancers such as chronic lymphocytic leukemia (CLL), this proportion is 2-3% (15). Chromosomal translocations and mutations can juxtapose proto-oncogenes with constitutively active promoters, leading to the deletion of tumor suppressor genes or the generation of chimeric oncogenes (16). Genomic sequencing of germline and somatic genomes has revealed the underlying pathological mechanisms of OS and syndromes with genetic susceptibility to OS. For example, a study on genomic alterations in childhood cancers showed that OS exhibits the highest frequency of Somatic Variant (SV) among all childhood cancers (17). The TP53, RB1, ATRX and DLG2 genes are frequently altered by SVs and/or Single - Nucleotide Variants (SNVs) in OS. In one study, among 19 patients with OS, tumor suppressor p53 was detected to be inactivated by translocation to the first intron of the TP53 gene in 9 cases. Although SNVs in the OS genome are relatively uncommon, both SVs and SNVs can lead to inactivating mutations in the p53 pathway, which is a feature found in 95% of OS (13). Studies have shown that the incidence of OS is 0.0003%. The incidence is relatively high among adolescents (0.8-1.1/100,000 per year between ages 15–19 years old) (18, 19). In young patients, bone OS mainly corresponds to the extremities, while the number of axial tumors increases with age (20).

Standard OS treatment includes surgery and chemotherapy. When combined treatment (neoadjuvant chemotherapy, surgery and adjuvant chemotherapy) is used, the 5-year survival rate for patients without metastatic disease at diagnosis is 60%-80% (21, 22). However, for patients with poor response to chemotherapy and patients with metastatic disease, the prognosis is much worse, with survival rates of <50% and <30%, respectively (23, 24). Although the comprehensive treatment plan of OS has been greatly improved, the overall survival rate of patients is still only 60% (25).

### 2.2 Molecular pathological mechanism

Multiple signaling pathways are involved in the development of OS and other types of cancers. OS is characterized by a high degree of genetic instability, which may hinder the understanding of its pathogenesis. Due to the dysregulation of the cell differentiation process, OS develops from osteoblasts and even more commonly from pluripotent precursor cells.

#### 2.2.1 Wnt signaling pathway

The Wnt signaling pathway is crucial for cell proliferation and differentiation. The Wnt pathway upregulates proliferation-stimulating oncogenes such as c-Myc, CCND1, and c-MET. For example, research has found that isoquercitrin (ISO) significantly inhibits proliferation, induces EMT migration, and induces apoptosis of OS cells *in vitro*. *In vivo* experiments, it was found that ISO exerts its anti-tumor effect partly by inhibiting the wnt/β-catenin signaling pathway (26). CGREF1 regulates the proliferation of OS cells *in vitro* and *in vivo* by regulating the wnt/β-catenin signaling pathway (27).

#### 2.2.2 FoxO signaling pathway

The FoxO signaling pathway is responsible for cell cycle regulation and apoptosis. This pathway can be activated in the wnt signaling cascade. For example, compared with osteoblasts, OS cells express more fucosyltransferase 4 (FUT4). Inhibition of FUT4 expression significantly inhibits the proliferation, invasion and migration abilities of OS cells, and also increases the apoptosis of OS cells. The wnt/β-Catenin signaling pathway is blocked by up-regulating FOXO1 expression, and then FOXO1 expression is inhibited by inhibiting FUT4 expression (28).

#### 2.2.3 PI3K/Akt/mTOR pathway and MAPK pathway

The PI3K/Akt/mTOR pathway and the MAPK pathway play crucial roles. For example, through transcriptome sequencing, it is found that the expression of HMGCL is down-regulated in OS and is related to the prognosis of OS patients. Overexpression of HMGCL can inhibit the activation of the PI3K/Akt/mTOR signaling pathway mediated by β-hydroxybutyrate (β-HB), thereby suppressing the proliferation, migration, and invasion abilities of OS cells, and simultaneously inducing an increase in the level of autophagy (29). Treatment of transfected OS cells and osteoblasts with corylin (0, 2.5, 5, 10, 30 μM) revealed that corylin inhibits the migration and invasion of OS cells by regulating the p38 MAPK signaling pathway (30).

#### 2.2.4 Notch signaling pathway

The Notch signaling pathway is a key pathway for controlling cell differentiation and preservation, including osteoblast differentiation and preservation of osteoblast stem cells (31). For example, cell migration-inducing protein (CEMIP) can promote the proliferation and metastasis of osteosarcoma cells by triggering the activation of the Notch signaling pathway. When the expression of the CEMIP gene is inhibited, the expression levels of Notch signaling pathway-related proteins (such as Jagged1 and Hes1) and the degree of signal activation show a downward trend both *in vivo* and *in vitro (* 32). Microfibrillar-associated protein 2 (MFAP2) is significantly associated with the Notch1 pathway in OS. Its elimination inhibits the expression of the Notch1 protein. In addition, Notch1 activation reverses the inhibitory effect of MFAP2 knockdown on the malignant characteristics of U2OS cells (33).

#### 2.2.5 NF-κB pathway

The NF-κB pathway is responsible for cell proliferation, prevention of apoptosis, and transcriptional regulation of various genes in response to injury factors and cytokines, and plays a crucial role in the pathogenesis of OS (34). For example, baicalein (SCU) blocks OS growth by inhibiting TLR4 expression and disrupting the TLR4-TRAF6 interaction, leading to NF-κB inactivation, showing a dual effect (35). The FN14 carried by bone marrow mesenchymal stem cells (BMSC) activates the NF-κB pathway to trigger PANoptosis in OS cells and significantly improves the long-term survival rate of mice (36).

#### 2.2.6 p53 gene

Another important mechanism is the p53-dependent pathway, which is involved in DNA damage response, cell cycle arrest, apoptosis, and tumor suppression. For example, c-Myc (Myc) directly upregulates γ-glutamylcyclotransferase (Ggct) by binding to its promoter, and deletion of the Myc binding site by genome editing weakens the tumorigenic potential of p53-deficient OS cells (37). As a bacterial β-lactamase inhibitor, clavulanic acid can bind to the LACTB protein and block its function, thereby inhibiting the proliferative ability of OS cells. It also has a dual regulatory effect on wild-type p53 (WT-p53) and mutant p53 (38).

**Figure 1:**
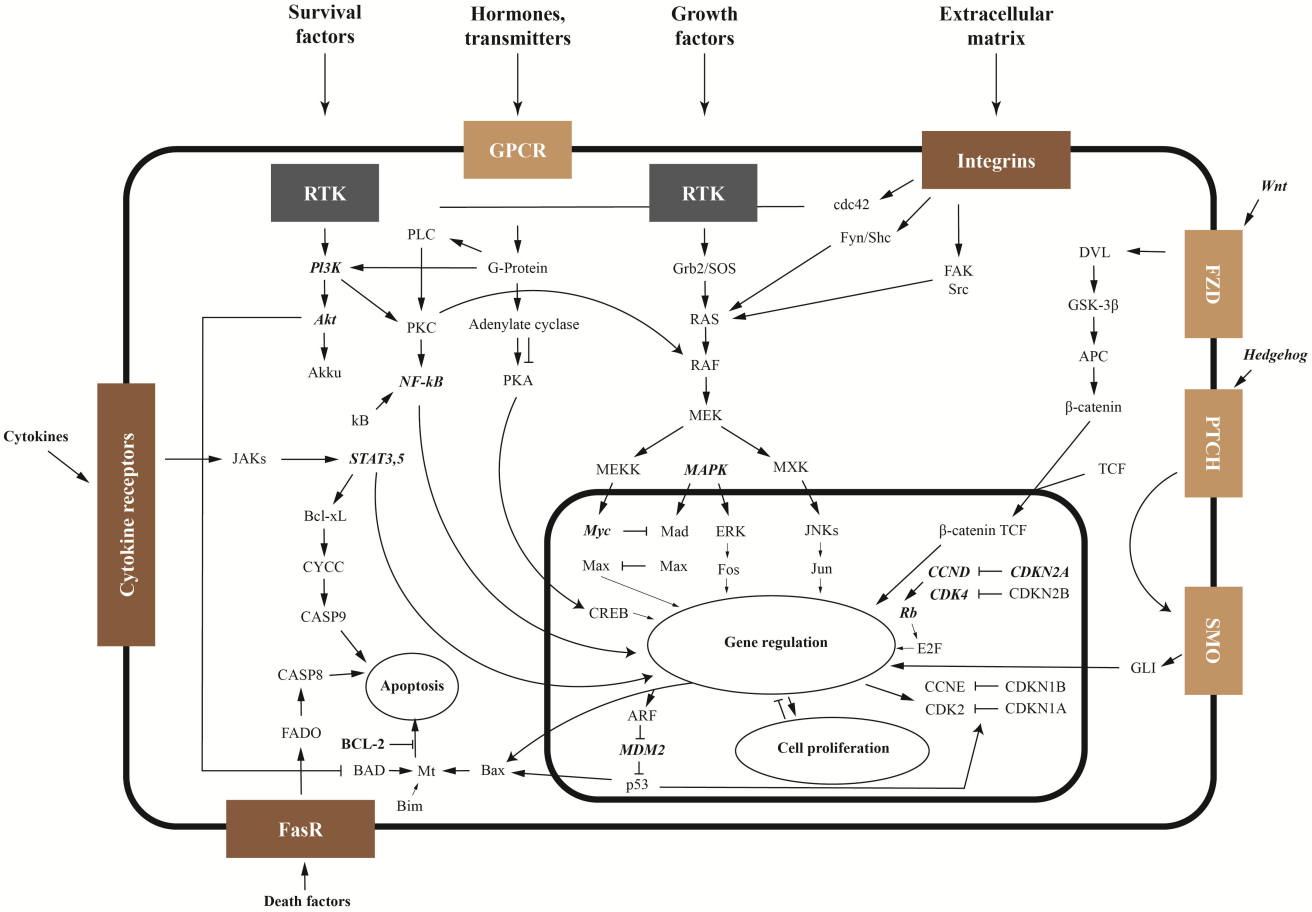
Signaling pathways and key genes in sarcoma (key genes are shown in bold italics).

The balance between tumor cell apoptosis and survival depends on the activities of anti-apoptotic proteins Bcl-2, Wnt and NFκB signaling pathways, as well as the ratio between the activities of mitogen-activated protein kinase (MAPK) and phosphatidylinositol-3-kinase/protein kinase B (PI3K/Akt) signaling pathways. The main signaling pathways and mechanisms of OS pathogenesis are shown in **Figure 1** Another important proliferation factor is the multifunctional protein YBX1. After secretion, YBX1 can stimulate cell migration and proliferation.

At present, the treatment methods for OS mainly include surgical resection, systemic chemotherapy, and targeted radiotherapy. Chemotherapy drugs such as doxorubicin and paclitaxel are widely used clinically (39), but there are problems such as difficult - to - solve recurrence and metastasis, and chemotherapy resistance reducing the efficacy. Molecular targeted therapy has high specificity and involves molecules such as CD44 and MMPs, and signaling pathways such as Notch and Wnt (40, 41). However, chemotherapy is still irreplaceable, and the Methotrexate, Doxorubicin, Cisplatin (MAP) regimen is the first - line recommended chemotherapy regimen (18).

### 2.3 The core role of CSCs in osteosarcoma

#### 2.3.1 The relationship between tumor stem cells and drug resistance and metastasis

CSCs in OS possess stemness properties and chemotherapy resistance and are one of the key reasons for poor chemotherapy response. CSCs exhibit several characteristics that may confer chemotherapy resistance, such as the abnormal expression of ATP-binding cassette proteins, activation of DNA repair capabilities, and overactive apoptotic regulatory elements (42). For example, it is found that miR-197-3p confers stemness and chemoresistance to OS by targeting SPOPL (43). Knockout of Ring Finger and WD Repeat Domain 3 (RFWD3) significantly reduces chemotherapy resistance of OS, In addition, GA can regulate apoptosis-related proteins (such as cPARP, Bcl-2 and Bax), thereby inhibiting angiogenesis and inducing DNA damage and apoptosis (44).

The clinical treatment of metastatic OS faces severe intratumoral heterogeneity, with its overall survival rate being less than 20% (45). The dedifferentiation process of tumor cells is an important factor driving the growth and metastasis of OS (46). The MSC markers CD117 and Stro-1 are double-positive expressed in some human and mouse OS cells, and these cells exhibit stronger multi-lineage differentiation potential, invasive ability, and metastatic tendency. The abnormal expression of the dedifferentiation marker CD133 is significantly associated with lung metastasis and poor prognosis in osteosarcoma patients (47). Through mRNA sequencing and functional analysis, THBS1 and ITGAs were identified as key molecules for dedifferentiation. Among them, THBS1 accelerates the dedifferentiation process and enhances the lung metastasis ability by promoting cytoskeleton remodeling, while ITGA1 and ITGA6 play important roles in mediating the transmission of extracellular signals into the cell (48). However, current research on the roles of CSCs in osteosarcoma drug resistance and metastasis faces limitations. On the one hand, most of the mechanism studies are still at the cell and animal model levels, and the specific roles and regulatory networks in human OS still require more in-depth clinical verification. On the other hand, although some key molecules such as THBS1 and ITGAs have been screened out, the research and development of specific intervention methods for these targets lags behind, making it difficult to rapidly translate into clinical treatment plans. How to break through the barriers between basic research and clinical application remains a key issue that urgently needs to be solved.

#### 2.3.2 Molecular mechanism of stemness maintenance

The wnt/β-catenin signaling pathway is usually in an inactive state in normal bone cells and highly differentiated cancer cells. Its signal transduction depends on the interaction between the secreted glycoprotein ligands and the signal-receiving cells. This pathway is closely related to tumors derived from epithelium and mesenchyme. Besides leukemia, colon cancer, breast cancer, and prostate cancer, it is also associated with bone tumors (49). In view of this, the development of drugs targeting the Wnt pathway has great potential. Relevant drug types include antibody-based therapies, vitamin D derivatives, small molecule inhibitors, and non-steroidal anti-inflammatory drugs (NSAIDs) (50, 51). For example, the small molecule compound boldine can activate the wnt/β-catenin pathway in human urine-derived stem cells (hUSCs), thereby promoting cell proliferation and multi-directional differentiation (52). Semaphorin 3A (Sema3A) activates the wnt/β-catenin pathway to protect against hydrogen peroxide-induced oxidative stress damage and repair the impairment of osteogenic differentiation function in periodontal ligament stem cells (PDLSCs) (53).

Notch signaling, as a key developmental pathway regulating stem cell self-renewal, has final effects that can be influenced by the differentiation state of somatic stem cells and crosstalk with other signaling pathways (54, 55). The Notch pathway is involved in regulating CSCs in various malignant tumors such as medulloblastoma, glioblastoma, and pancreatic cancer. Currently, multiple drugs targeting components of the Notch pathway have entered the clinical trial stage and have great potential in overcoming CSCs drug resistance (56). For example, NOTCH1 signaling affects the maintenance of MSCs stemness and the chondrocyte differentiation process by regulating the EZH2 protein (57); in hepatocellular carcinoma (HCC), CD146 upregulates the expression of JAG2 by activating the NF-κB signal, thereby activating the Notch pathway, leading to enhanced tumor cell stemness and chemotherapy resistance. Moreover, overexpression of JAG2 can restore the Notch signaling activity and stemness phenotype caused by CD146 knockdown (58).

**Figure 2:**
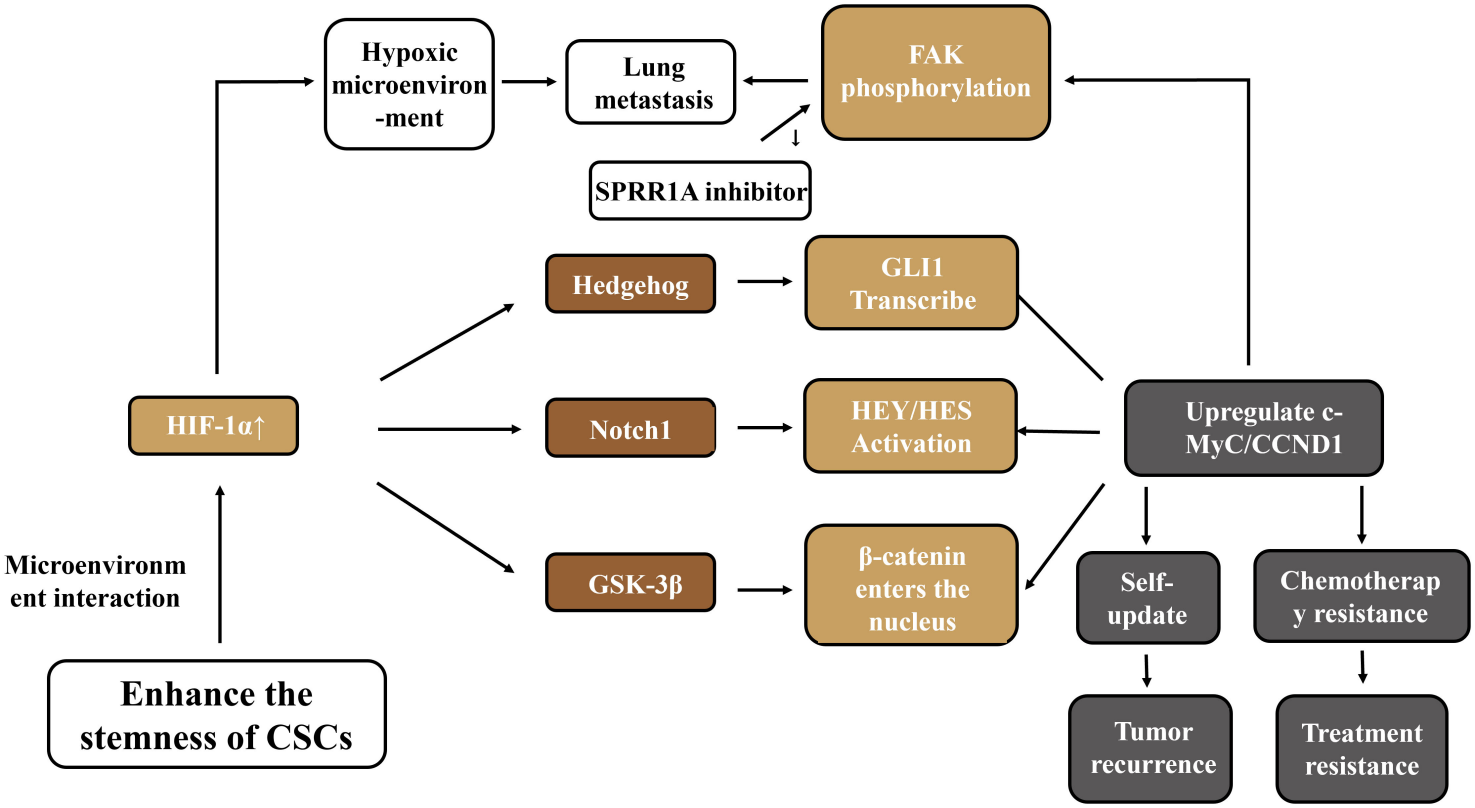
Mechanisms of CSCs in regulating osteosarcoma.

As an important regulatory pathway in embryonic development, the Hedgehog signaling pathway is involved in cell differentiation, proliferation, and self-renewal processes, and plays a role in maintaining the homeostasis of adult cells and tissues as well as the renewal of stem cells (59). This signaling pathway can collaborate with other pathways to promote the invasive ability of OS. Natural compounds such as lactoferricin can block the Hedgehog signal by inhibiting the activity of GSK3-β, thereby reducing the migration, invasion, and metastasis abilities of tumor cells (60). In disease models, the imbalance of osteogenic-adipogenic differentiation in steroid-associated avascular necrosis of the femoral head (SANFH) is related to IFT80 inhibiting adipogenic differentiation and promoting osteogenic differentiation of BM-MSCs by activating the Hedgehog pathway (61). In hepatocellular carcinoma (HCC), 3-hydroxy-3-methylglutaryl-coenzyme A reductase (HMGCR) promotes the regeneration and metastasis of tumor cells by activating the Hedgehog signal (62). The Wnt/Notch/Hedgehog pathway constitutes the core network for regulating the stemness of CSCs. Targeted intervention (such as AZD1080 inhibiting GSK-3β) can reverse drug resistance. However, the cross-regulation among pathways leads to the easy occurrence of compensatory activation in single-targeted therapy. Moreover, the dominance of pathways varies in different subtypes of OS. There is an urgent need to develop a combined blockade strategy based on molecular typing. To more systematically visualize the multi - dimensional regulatory network of cancer stem cells (CSCs) in maintaining stemness, generating treatment resistance, and promoting tumor progression in osteosarcoma, as shown in **Figure 2**, the pathway interactions and functions of key molecules described in this section were integrated to intuitively present how core pathways such as Wnt/β - catenin, Notch, Hedgehog, and related molecules synergistically support the malignant biological characteristics of CSCs.

#### 2.3.3 Immunosuppressive role of OS-specific CSCs

CSCs drive immune escape in OS by secreting immunosuppressive molecules and remodeling the microenvironment, and are key factors in the immunosuppression of the OS microenvironment. They are highly heterogeneous. Genes in the stemness maintenance and differentiation subgroups are involved in the communication with tumor-associated macrophages (TAMs). For example, RARRES2 plays a role in the intercellular communication between OSCs and TAMs, and insulin-like growth factor 1 secreted by TAMs can promote the stemness maintenance of OSCs mediated by RARRES2. This interaction is jointly involved in the immunosuppressive process of the microenvironment (63). At the same time, CSCs participate in immune escape by regulating the immune checkpoint molecule PD-L1, and key CSC-related genes such as MEF2C, SPI1, and MYC play specific roles: some genes affect the infiltration of effector T cells by regulating the chemotaxis or activation signals of immune cells, and some indirectly promote the secretion of immunosuppressive factors such as TGF-β and IL-6 by enhancing the stemness of CSCs, exacerbating the inhibitory state of the microenvironment (64).

However, the elevated heterogeneity of CSCs poses a huge challenge to the treatment of OS. There are significant differences in the mechanisms of promoting metastasis and immunosuppression among different sub-populations of CSCs. Some sub-populations may mainly promote metastasis by remodeling the microenvironment, while others focus on achieving immune escape by regulating immune checkpoint molecules or secreting inhibitory factors. The diversity of these mechanisms makes single-targeted therapy difficult to be fully effective. How to accurately distinguish and specifically intervene in different functional sub-populations remains a treatment bottleneck that urgently needs to be overcome.

## 3 Essentials of stem cell biology

### 3.1 Classification and characteristics of stem cells

Stem cells are a type of undifferentiated cells that exist in embryonic, fetal, and adult stages. They are the growth foundation of tissues and organs and have the characteristics of self-renewal, cloning, and multi-directional differentiation (**Table 1**). They have a significant role in the promotion of the repair and regeneration of damaged tissues (65).

**Table 1:** Summarizes the biological characteristics of ESCs, MSCs and iPSCs.

Stem cell types	Source	Differentiation potential	Ethical restrictions	Clinical application challenges
ESCs	Inner cell mass of blastocyst	Totipotency	Strict ethical restrictions	Risk of immune rejection, limited to basic research (66, 68)
MSCs	Bone marrow, fat, umbilical cord, etc.	Multidirectional differentiation	No ethical controversy	Batch heterogeneity (74, 75)
iPSCs	Somatic cell reprogramming (factors such as Sox2/Oct3/4)	pluripotent (similar to ESCs)	Potential carcinogenic risk of viral vectors	The differentiation efficiency is low (79–81)

#### 3.1.1 Embryonic stem cell

Embryonic stem cells (ESCs) are taken from the inner cell mass of blastocysts. A blastocyst is a hollow sphere mainly composed of cells. In humans, blastocysts form 3 to 5 days after the fertilization of an egg cell and a sperm. During normal development, the cells in the inner cell mass will differentiate into more specialized cells and then form the entire body, that is, all the tissues and organs of the human body.

ESCs have the potential to generate all cell types in the body, but are subject to strict ethical and scientific limitations in basic research and clinical translation. The International Society for Stem Cell Research (ISSCR) emphasizes strict review of research on organoids, embryos, embryo models based on stem cells, chimeric embryos, and genome editing (66, 67). The ISSCR stipulates restrictions on embryo model-related research based on stem cells within 14 days (“14-day rule”), that is, researchers should not culture human embryos *in vitro* for more than 14 days (calculated from the date of fertilization). Because on the 14th day, the Primitive Streak begins to appear in the fetal development process and the body axis of the embryo begins to be established. Cultivation that exceeds 14 days may form a potential and viable life form. Currently, the “14-day rule” has been widely accepted by researchers and research funders in many countries. For example, in countries such as Germany and Austria, any research on human embryos is illegal; in other countries, such as the United Kingdom, Japan, Australia and Canada, the “14-day rule” has been legislated. In the United States and Israel, there is no law explicitly prohibiting or restricting human embryo research, but these studies cannot be funded by the federal government in the United States (66, 68). Although ESCs have the potential for totipotent differentiation, their clinical applications have always been limited by ethical controversies and the rigid constraints of the “14-day rule”, and the risks of chimeric embryos in cross-species research have not been fully clarified. These factors make it difficult for them to become the core choice of conventional treatment methods.

#### 3.1.2 Adult stem cells

Adult MSCs are distributed in different tissues and can be isolated and expanded from bone marrow fat, skeletal muscle, peripheral blood, liver, skin, lung tissue, dental pulp, and neonatal-related placenta, amnion, and umbilical cord tissue (69, 70). MSCs are the most representative and widely used adult stem cells in translational research. Different types of MSCs have unique therapeutic mechanisms for treating diseases. MSCs have the potential to differentiate (trans-embryonic layer differentiation) into tissue-specific cells of different types. At the same time, they have paracrine functions and immune regulation and other characteristics (71, 72). In recent years, people have used MSC to treat different types of diseases, such as autoimmune diseases, cardiovascular diseases and have achieved certain curative efficacy (73).

With the advancement of basic research and clinical application of MSCs, it is found that MSCs encounter many problems to be solved in the process of clinical transformation. First, MSCs therapeutic products derived from autologous or allogeneic tissues have heterogeneity (difference) between batches and within batches, which hinders large-scale production of uniform and stable cell products. SCs derived from allogeneic tissues inherently lack the capacity for personalized autologous therapy. Second, MSCs products prepared through processes such as tissue isolation, purification, and amplification cannot obtain the minimum stem cell dose required for a single treatment at a low passage number. Continuous amplification *in vitro* is required, but after continuous amplification *in vitro*, difficulties arise in terms of avoiding stem cell senescence, loss of stemness, chromosomal variation, and epigenetic changes. In addition, the functionality and limitations of MSCs treatment have also reached a consensus. Although infusion of MSCs (autologous or allogeneic) is safe, it is also found that its curative effect is not strong and often inconsistent (74, 75). Although mesenchymal stem cells perform outstandingly in terms of safety, issues such as individual differences in their efficacy, batch instability, and the decline in stemness caused by *in vitro* expansion have exposed their core weaknesses in large-scale and standardized clinical applications. It is necessary to break through technical bottlenecks to achieve a leap in therapeutic value.

#### 3.1.3 Induced pluripotent stem cells

The renowned Japanese stem cell researcher Shinya Yamanaka published a series of seminal papers (76, 77). Concentrate on researching the reprogramming and induced differentiation of mouse and humanized somatic cells into Induced Pluripotent Stem Cells (iPSCs), realizing almost the same proliferation and differentiation potential of iPSCs and ESCs. Thus, getting rid of the fatal ethical issues faced by ESCs research (using “embryos”) and opening up a new way for stem cell translational medicine research to treat diseases. Using both viral (76, 78) and non-viral (77) vectors, Yamanaka demonstrated that the forced expression of key transcription factors (Sox2, Oct3/4, Klf4, c-Myc, and Nanog) could reprogram mouse and human somatic cells to dedifferentiate and generate iPSCs. His research found that like ESCs, iPSCs still retain their good biological characteristics and potential after being expanded *in vitro* for many generations (proliferating millions of times) (79–81).

The basic clinical transformation research of iPSCs is increasingly being conducted, and there are also more and more clinical transformation studies on the differentiation of iPSCs into other functional cells (**Table 1**). Animal experiment studies have shown that the directional differentiation of iPSCs into cardiomyocytes has allowed to achieve curative effects in repairing myocardial ischemia (82). In animal models of muscle atrophy (duchenne muscular dystrophy, DMD), the use of human iPSCs to induce and differentiate into muscle progenitor cells (MuPCs) for intervention treatment can effectively repair muscle damage and atrophy (83). iPSCs are reprogrammed through animal and human (skin) epithelial cells. Products of MSCs derived from iPSCs have achieved good curative effects in different animal models. In mouse limb ischemia models, muscular dystrophy models, trauma models, inflammation models, bone defect models and tumor models, good clinical transformation effects have been achieved (84–87). Although induced pluripotent stem cells circumvent the ethical dilemmas of embryonic stem cells, the potential genetic mutation risks during the reprogramming process, the cell type-dependence of differentiation efficiency, and the time-consuming preparation process in autologous transplantation are still key obstacles restricting their transition from the laboratory to the clinic.

### 3.2 Tumor stem cell theory

Research has found that the origin of OS cells is closely related to MSCs (88). Since the 1970s, new adjuvant and postoperative adjuvant chemotherapy regimens have been carried out clinically for OS. However, the survival rate of metastatic patients has not been significantly improved over the years. Therefore, understanding the molecular mechanism of the interaction between MSCs and OS cells has become a key research direction for improving treatment efficacy. The tumor microenvironment (TME) plays an important regulatory role in the functional activation of tumor cells (89–91). Among them, activated cancer-associated fibroblasts (CAFs), as a key non-tumor cell component in the TME, play an important role in tumor development by secreting cytokines that promote immune escape and remodeling the extracellular matrix (92, 93). Research shows that MSCs may be involved in the invasion and progression of OS (94), and osteosarcoma cells can induce the differentiation of bone marrow MSCs into CAFs in a co-culture system (95). Although the phenomenon that bone marrow MSCs promote tumor progression through fibroblast differentiation has been confirmed, the specific induction mechanism of their transformation into CAFs remains unclear (96, 97).

Among the transcription factors that regulate the differentiation of MSCs, transforming growth factor-β (TGF-β) is an important tumor-promoting factor by promoting tumor growth, inducing epithelial-mesenchymal transition (EMT), and enhancing invasion ability. There is evidence showing that TGF-β1 can mediate the differentiation of MSCs into CAFs in colorectal cancer, and OS cells can also induce the CAF-like transformation of MSCs by secreting TGF-β1 (98–100). In addition, methyltransferase-like 3 (METTL3) is highly expressed in osteosarcoma cells (101, 102), and the N6-methyladenosine (m6A) modification it participates in can act on the methylation sites of TGF-β1 mRNA, thus affecting related signaling pathways (103, 104).

### 3.3 Function and application of MSCs

#### 3.3.1 Homecoming effect

As an important mechanism of cell migration, cell homing has a significant impact on organism development, tissue regeneration, and disease progression. This concept was first used to describe the property of lymphocytes in circulating blood migrating to their origin sites such as lymph nodes, and its biological behavior is similar to the homing instinct of birds (105). In 2010, the Saito team proposed that under the action of specific stimulating factors, quiescent MSCs can be activated and migrate to damaged tissues, and then differentiate to replace damaged cells (106). This directed migration ability of stem cells is likened to an *in-vivo* navigation system, which can autonomously locate the tissue repair site and promote timely regeneration.

MSCs naturally possess the characteristic of migrating towards various chemokine signals secreted by tumor tissues or their microenvironments, enabling them to target and colonize tumor lesions. This unique property has prompted MSCs to become a new type of active carrier for delivering anti-tumor drugs and genetic materials. The delivery system constructed based on MSCs can achieve the targeted transport of chemotherapeutic drugs (such as doxorubicin, paclitaxel, etc.), effectively solving the problems of short half-life of traditional drugs and insufficient tumor specificity (107, 108). For example, in early acute respiratory distress syndrome (ARDS), primed MSCs show enhanced homing ability, improved lung function and reduced inflammation (109). The non-living tumor homing vector of mitoxantrone (MTX), CT-MTX, is a new type of non-proliferative drug delivery platform. It combines the tumor homing ability of MSCs with enhanced safety and controlled release properties, and induces immunogenic cell death (ICD) of prostate cancer and other immunologically “cold” tumors to improve immune infiltration (110). *In vitro* studies have shown that the migration ability of BMSCs pretreated with ELABELA (ELA) is significantly enhanced under hypoxia-reoxygenation (H/R) conditions. This not only improves the homing efficiency of BMSCs to the site of myocardial injury, but also significantly enhances their ability to repair myocardial injury *in vivo (* 111).

#### 3.3.2 Immune regulation

MSCs can efficiently mediate the targeted delivery of therapeutic genes through genetic engineering techniques, including tumor-killing genes and immune system regulatory genes. The mechanism is closely related to the specific expression of therapeutic genes at the tumor site to achieve tumor suppression or killing effects (112). MSCs and their secreted proteome can play a key role in biological processes such as immunomodulation, anti-inflammatory response, angiogenesis promotion, and anti-fibrosis by balancing pro-inflammatory and anti-inflammatory signals in different diseases (113, 114).

In terms of immune cell regulation, MSCs transplantation can lead to an increase in the proportion of CD4+FOXP3+ regulatory T cells (Tregs) in peripheral blood monocytes, while the proportion of Tregs within the B cell population shows a downward trend (115). At the same time, the study found that UC-MSCs can promote the proliferation and inhibit the apoptosis of CD1c^+^ dendritic cells (DCs) by secreting FLT3 ligand (FLT3L) (116). Clinical follow-up data show that the level of natural killer T cells (NKT cells) following transplantation of UC-MSCs was significantly lower than the baseline during the 18-month observation period (117).

From the perspective of cytokine regulation mechanisms, TGF-β and interleukin 10 (IL-10) are involved in the proliferation and differentiation process of Treg cells. After MSC treatment, the concentration of TGF-β increases, but the change of IL-10 is not significant (118). In kidney diseases, the secretome of MCSs can mediate innate and adaptive immune responses through cell-to-cell contact, cytokines or regulatory factors (119).

MSCs promote the proliferation of pancreatic islet β cells and improve hyperglycemia through the PI3K/Akt signaling pathway (120). And promote islet growth by reducing the effects of IL-1 and TNF-α (121). UC-MSCs can also improve blood glucose levels and protect endothelial cells from hyperglycemia damage through the paracrine effect mediated by the MAPK/ERK signaling pathway (122).

## 4 Dynamic interactions and paradoxical effects of stem cells in the microenvironment

TME, which includes immune cells, peritumoral vasculature, fibroblasts, various signaling molecules and the extracellular matrix (ECM), plays an important role in tumor progression (123). Immune cells are key components of TME. Monocytes and macrophages are the most predominant (>40%) immune cells in the TME (124). In addition, monocytes and macrophages account for 70-80% of the total number of tumor-infiltrating myeloid cells (125). It is worth noting that macrophages in the TME exhibit a high percentage of M2 polarization, which may accelerate the malignant progression of OS. The interaction between tumor cells and immune cells in the TME supports tumor progression. Tumor cells can recruit and reprogram monocytes and macrophages through cell-cell contact or paracrine signaling to remodel the vasculature and ECM (126). In turn, extracellular vesicles (EVs) released by tumor cells play an important role in coordinating TME and mediating the interaction between tumor cells and immune cells (127). The immunomodulatory ability of MSCs makes them an ideal drug carrier (such as the IDD-1040-paclitaxel complex). However, the secretion of factors such as TGF-β can promote the differentiation of CAFs and accelerate immune escape. The key contradiction lies in the inability to precisely control the functional polarization of MSCs in the TME. In the future, it is necessary to develop a microenvironment-responsive “intelligent” MSC engineering platform to avoid the risk of tumor promotion.

### 4.1 The “double-edged sword” effect of MSCs on tumors

MSCs are a type of precursor cells with self-renewal and multi-directional proliferation potential. Under specific conditions, they can differentiate into various mesenchymal lineage cells (128, 129). They have a wide range of sources, including bone marrow, adipose tissue, skin, salivary glands, limb buds, dental tissues, menstrual blood, and placenta. The isolation of MSCs is usually based on their plastic adherence characteristics, and the main methods include enzymatic digestion and tissue block culture (explant technique). The explant method requires rinsing the tissue and cutting it into small fragments, which are then inoculated into a culture container containing growth medium. The enzymatic digestion method uses enzymes that degrade the extracellular matrix to treat tissue blocks to obtain single cells (130, 131). These cells highly express specific adhesion molecules on their surface, such as CD13, CD44, CD73, and CD166. Moreover, MSCs from different tissue sources have specific markers. For example, placental MSCs highly express CD29 and CD49b, while the expression level of CD90 in bone marrow MSCs is significantly higher (132).

The immunomodulatory function of MSC is achieved through the secretion of various cytokines, including transforming growth factor (TGF-β), prostaglandin E2 (PGE2), indoleamine 2,3-dioxygenase (IDO), nitric oxide (NO), etc (133–135).

According to functional differences, MSCs can be divided into multiple subtypes. Among these, MSC1 and MSC2 represent two key phenotypes: MSC1 exhibits pro-inflammatory properties and primarily exerts anti-tumor effects, whereas MSC2 demonstrates immunosuppressive functions and may promote tumor growth. This two-way regulatory effect on tumor development is achieved through different mechanisms: the pro-tumor effects include the secretion of growth factors, the promotion of tumor angiogenesis, and the construction of the tumor stem cell microenvironment (136), the anti-tumor effects involve pathways such as the activation of the immune response, the inhibition of angiogenesis, the regulation of cell signaling pathways, and the induction of cancer cell apoptosis (137).

### 4.2 Cooperative regulation of immune cells and stem cells

In the TME, macrophages, as the innate immune cell population with the largest number of infiltrations, not only play a key role in anti-tumor immunity but also possess antigen-presenting functions to activate the adaptive immune response. This type of immune cell exhibits significant phenotypic heterogeneity and functional plasticity. It can undergo phenotypic polarization in response to different microenvironmental stimuli, presenting a continuous functional spectrum ranging from anti-tumor to pro-tumor. According to differences in activation states and biological functions, macrophages are mainly divided into classically activated M1 type (pro-inflammatory phenotype) and selectively activated M2 type (anti-inflammatory phenotype). Together with mesenchymal stem cells (MSCs) in the TME, they form a two-way regulatory network for tumor development (138).

An increasing amount of evidence indicates that tumor-associated MSCs can induce the polarization of macrophages into the M2 type, enhancing the tumor immunosuppressive microenvironment. Research has found that exosomes derived from TAMSCs infiltrating into the TME of human breast cancer can induce the differentiation of monocytic-myeloid-derived suppressor cells (M-MDSCs) into M2 macrophages, promoting tumor immune escape. At the same time, it can also enhance the epithelial-mesenchymal transition (EMT) of breast cancer cells, facilitating tumor metastasis (139). When macrophages were co-cultured with BMMSCs, it was found that CXCL-12 derived from BMMSCs could induce M2-type polarization of macrophages and enhance the immunosuppressive microenvironment (140). TAMSCs infiltrating into melanoma tumors can promote their high secretion of milk fat globule epidermal growth factor 8 (MFG-E8) under the tumor hypoxic microenvironment, inducing M2 polarization of macrophages (141).

The IL-17 accumulated in TME can promote the generation of inducible-nitric-oxide-synthase (iNOS) in TAMSCs, upregulate the programmed cell death 1 ligand 1 (PD-L1) on the surface of TAMSCs, inhibit the normal function of T cells, and promote tumor immune escape (142). In addition, TAMSCs can reduce the efficacy of anti-PD-L1 immune checkpoint blockade by secreting CX3CL1, CCL2 and TGF-β (143).

### 4.3 Hypoxia and metabolic reprogramming

Hypoxia-inducible factor-1α (HIF-1α) can promote the continuous differentiation of cells under hypoxic conditions, promote angiogenesis, and maintain the stemness of CSCs (144). The increase in HIF-1α expression in BM-MSCs in response to breast tumor cells under normoxic conditions is mediated by ROS and JAK/Stat3, and both HIF-1α-dependent and -independent mechanisms increase the expression of VEGF in BM-MSCs to promote the angiogenic sprouting ability of endothelial cells in a VEGF-dependent manner (145). In addition, under hypoxic conditions, the effects of hBMSC-MVs on the progression of U2OS cells and tumor growth are related to the PI3K/AKT and HIF-1α pathways (146). Under hypoxic conditions, adipose-derived mesenchymal stem cells (AMSCs) *in vitro* have better viability, significantly downregulate the expression of inflammatory factors, alleviate macrophage inflammation, and activate the PI3K/AKT/HIF-1α pathway (147). The use of ultrasound-targeted microbubble destruction can increase the migration of mesenchymal stem cells and promote cartilage repair in rats through the HIF-1α-mediated glycolytic pathway (148).

## 5 Treatment and challenges of OS based on stem cells

In recent years, a variety of emerging strategies have been developed based on stem cell biology for the treatment of OS (**Table 2**). These strategies aim to target the drug resistance of CSCs, utilize the tumor-homing properties of mesenchymal stem cells (MSCs) to deliver therapeutic payloads, or reshape the immune response through gene editing technologies. However, challenges such as the heterogeneity of CSCs, the functional plasticity of MSCs, and the *in vivo* delivery efficiency have restricted clinical translation. The following will systematically review the mechanisms of action, representative regimens, and translational bottlenecks of four core strategies, providing a multi-dimensional perspective for overcoming treatment resistance.

**Table 2:** Comparison of treatment strategies related to osteosarcoma stem cells.

Treatment category	Mechanism of action	Represent drugs/methods	Advantages	Challenge
CSCs-targeted drugs	Inhibit stemness-maintaining pathways to induce differentiation or apoptosis of cancer stem cells (CSCs)	AZD1080; SPRR1A; DNMTi/HDACi	1. Specifically target the core pathways of CSCs 2. Reverse chemoresistance 3. Significantly inhibit tumor sphere formation in preclinical studies	1. The heterogeneity of CSC leads to differences in therapeutic efficacy. 2. Single-pathway inhibition is prone to trigger compensatory activation. 3. Epigenetic drugs have the risk of off-target effects (152, 161)
Drug delivery mediated by MSC	Utilize the tumor-homing characteristics of MSC to deliver anti-cancer drugs or gene therapy vectors	IDD-1040-Paclitaxel Complex; Engineered MSC Delivery of IFN-β/Oncolytic Virus	1. Enhance tumor targeting 2. Prolong the drug half-life 3. Improve the immune microenvironment	1. Cell retention due to pulmonary first-pass effect 2. Decrease in stemness after in vitro expansion 3. Tumor-promoting risk (172, 173)
Gene Editing and Cell Therapy	CRISPR edits the antigen target of CSC to construct highly efficient CAR-T cells	CRISPR-engineered anti-CD133 CAR-T; Oncolytic adenovirus vector (oAd-SA)	1. Precisely eliminate the CSC population 2. Synergistically enhance the immune response 3. Significantly inhibit metastasis in pre-clinical settings	1. Poor penetration of solid tumors 2. Risk of cytokine storm 3. Antigen loss leading to drug resistance (152, 161)
Optimization of drug administration routes	Change the stem cell infusion route, avoid pulmonary entrapment, and enhance target tissue engraftment.	Intra-arterial injection (such as renal artery, pancreatic artery)	1. Increase the local cell concentration of the lesion. 2. Prolong the survival time in the body (>21 days). 3. Enhance tissue repair.	1. The operation is complex and invasive. 2. It is difficult to promote clinically. 3. The long-term safety needs to be verified (181, 186)

### 5.1 Drug development targeting CSCs

CSCs can self-renew, differentiate, and repopulate the entire heterogeneous cancer cell population, thus explaining cancer recurrence and metastasis (75). CSCs-related signaling pathways, such as the Hedgehog, Notch, and Wnt signaling pathways, have attracted great interest as therapeutic targets for CSCs (149). For example, pharmacological inhibition of the HH pathway in CSCs using small molecule inhibitors cyclopamine or SANT-2 suppresses adhesion, invasion, and migration. This treatment also significantly reduces cell surface expression of Sonic Hedgehog (SHH) but does not affect CD133 expression (150), The small molecule Wnt inhibitor ICG-001 effectively inhibits CRC stemness and metastasis by suppressing MEIS1 expression (151). The small molecule inhibitor ASR490 can significantly inhibit the growth of BCSCs (phenotypically ALDH^+^ and CD44+/CD24-) and breast cancer (BC) cells. At the same time, it weakens the colony-forming ability and mammosphere-forming ability of BCSCs and BC cells. Its mechanism of action is related to the targeted elimination of the Notch1 signaling pathway in BCSCs and BC cells, thus effectively inhibiting breast cancer tumor growth (152).

Aberrant epigenetic regulation is closely related to tumor cell occurrence of various diseases. The occurrence and development of cancer are jointly driven by genetic variations, changes in epigenetic modifications, and environmental factors. Epigenetic activation of oncogenes and epigenetic silencing of tumor suppressor genes are key mechanisms in tumor progression. Among them, abnormal functions of DNA methyltransferases (DNMTs), histone deacetylases (HDACs), and histone methyltransferases (HMTs) are common in many types of tumors, making them important targets for anti-tumor therapy (153, 154). The US Food and Drug Administration (FDA) has approved multiple types of epigenetic drugs (epi-drugs) for clinical use, including DNA methyltransferase inhibitors (DNMTis), histone deacetylase inhibitors (HDACis), and histone methyltransferase inhibitors (HMTis), with indications covering various malignant tumors such as myelodysplastic syndromes, lymphomas, multiple myelomas, and epithelioid sarcomas (155, 156). DNA methyltransferases (DNMTs) and H3K4 methyltransferases maintain cell-autonomous regulation of CSCs, confer chemoresistance, maintain cycling quiescence, and reduce the migration and proliferation of BCCs (157). Histone deacetylase inhibitors (HDACis), when used alone or in combination with conventional chemotherapy, regulate gene expression by inhibiting histone deacetylases, thereby exerting anti-tumor effects. They lead to cell-cycle arrest, induce programmed cell death, and transform cancerous T cells, which can trigger durable positive outcomes in individuals with PTCL (158). Recent studies have found that SPRR1A is a key target in the treatment of stem cell-related OS. The research shows that SPRR1A is abnormally upregulated in OS stem cells. By regulating pathways such as focal adhesion kinase (FAK) and cyclin-dependent kinase 2 (CDK2), it directly affects the migration, adhesion ability and proliferation activity of tumor cells, and is closely related to the poor prognosis, recurrence and metastasis of osteosarcoma (159). In addition, AZD1080, as a specific inhibitor of GSK-3β, is a potential candidate drug for targeted OS treatment. Its target is clear. It targets glycogen synthase kinase-3β (GSK-3β), a key molecule for maintaining the stemness of OS cancer stem cells (CSCs), and can specifically target osteosarcoma CSCs. It can significantly inhibit the *in vitro* characteristic of sphere formation of U2OS and 143B osteosarcoma cells, down-regulate the expression of OCT4 and SOX2, and at the same time inhibit the phosphorylation of GSK-3β itself and its downstream regulatory genes (HEY1, HES1, CyclinD1, β-catenin), blocking the stemness-maintaining signaling pathway mediated by it (160).

However, intratumoral heterogeneity (ITH) promotes tumor evolution, leading to tumor lethality and treatment resistance (161). ITH not only comes from the accumulation of mutations and clonal expansion, but also from large-scale chromosomal rearrangements (162). Research analysis shows that this phenomenon may be attributed to the significant upregulation of the expression levels of epithelial-mesenchymal transition (EMT)-related genes and metastasis-related genes in hybrid cells (163). Another study showed that the fusion of MCF-7 breast cancer cells with MSCs led to increased proliferation, migration, and drug resistance. Further research indicated that the hybrid cells reprogrammed tumor cell energy metabolism of tumor cells by upregulating glycolysis-related genes (164). The latest research has found that the hybrid cells formed by the fusion of bladder cancer cells and MSCs significantly upregulate the expression of PD-L1 through epigenetic mechanisms. Subsequently, by inhibiting the phagocytic function of macrophages and inducing an immunosuppressive microenvironment, the immune escape ability of tumor cells is enhanced (165).

Despite the progress in treatment, the prognosis for most patients with malignant tumors remains unfavorable, mainly due to the continuous evolution and recurrence of these tumors. The high heterogeneity and plasticity of tumors often reduce the efficacy of single-target therapies. Targeting cell fusion can limit tumor evolution and offers a promising new approach for significant progress in cancer treatment. Recent studies have investigated various strategies to inhibit cell fusion in tumors. For example, syncytin-1, a fusion-promoting protein involved in physiological and tumor-related cell fusion (166). The delivery of EGFR knockout plasmids to circulating malignant cells effectively inhibits the fusion between tumor cells and normal cells (167).

### 5.2 Mesenchymal stem cells as drug delivery carriers

As a powerful method to improve the efficacy of MSCs, bioengineering methods can expand the application of MSCs-based therapies, especially in anti-cancer treatments. MSCs have been modified to deliver interferons, interleukins, anti-angiogenic agents, pro-apoptotic proteins, pro-drugs, or oncolytic viruses, in order to directly induce tumor apoptosis or activate immune cells to combat tumors (168). Research has found that IDD-1040 is a novel anti-cancer chemical conjugate that combines lipoic acid with Paclitaxel (PTX). It exhibits prolonged circulation, effective tissue distribution, and reduced metabolite formation *in vitro*, and its anti-cancer efficiency is superior to that of using PTX alone (169). In addition, the genetically engineered oncolytic adenovirus (oAdV) vector pDC316-oAd-SA was modified to express the SIRPα-mIgG1Fc fusion gene. This vector can enhance the anti-tumor immune response by remodeling the tumor-associated macrophage (TAM) microenvironment. Moreover, the improved oAd-SA can significantly enhance the phagocytic function of macrophages and exhibit a more significant tumor regression effect in mouse tumor models (170).

Generate overexpression of interferon-β (IFNB, IFNB-iPSCs) through genetic engineering, so as to apply it to immunotherapy and overcome the adverse consequences caused by systemic administration (171). For example, MSCs primed with IFN-γ and IL-1β significantly enhanced the suppression of T cell activity, inhibited TNF-α, and increased the production of IL-10 in macrophages. Signaling pathway analysis confirmed that the efficacy could be enhanced by regulating NF-κB and TNF-α signaling. In early ARDS, primed MSCs exhibited enhanced homing ability, improved lung function, and reduced inflammation (109).

It is worth noting that during the treatment process, MSCs may differentiate into fibroblasts to promote fibrosis or facilitate metastasis through fusion. Safety remains the most concerning issue in the clinical application of MSCs. For example, *in vitro*, umbilical cord mesenchymal stem cells (UCMSCs) reduce melanin content and tyrosinase activity, inhibit the viability, proliferation, migration, and invasion of melanoma cells, and promote the apoptosis of melanoma cells (172). Moreover, the viral vectors used to introduce plasmid DNA may lead to insertional carcinogenesis, adverse immune responses, and increased production costs. Hazards can be systematically classified into acute complications (such as inflammatory reactions or embolic events), moderate complications (including graft-versus-host disease (GVHD) or secondary infections), and long-term complications (especially potential tumorigenesis). Considering the observed phenomenon that MSCs may promote rather than inhibit tumor proliferation, it makes MSCs an unstable alternative for treatment (173).

### 5.3 Gene editing and CAR-T cell therapy

Clustered regularly interspaced short palindromic repeats (CRISPR)/associated protein 9 (CRISPR/Cas9) has brought a revolutionary change to the field of gene therapy (174). In the context of cancer immunotherapy, this technology has been widely used in the screening of novel therapeutic targets (175), the analysis of drug resistance mechanisms, and the research and development of the new generation of chimeric antigen receptor T cells (CAR-T) (176, 177). For example, knockout of the CD47 gene by CRISPR/Cas9 RNA lipid nanoparticles can effectively inhibit the growth of mesenchymal glioblastoma *in vivo (* 178).

As a breakthrough in cancer immunotherapy, CAR-T therapy offers new treatment options for patients with relapsed and refractory malignancies. CAR-T cells are T lymphocytes that have been genetically engineered to specifically recognize and eliminate tumor cells (179). Research shows that anti-CD133 CAR-T cells exhibit significant killing efficacy against CD133-positive BGC-823 cells treated with cisplatin, accompanied by the up-regulation of activation markers and the massive secretion of cytotoxic cytokines. In addition, the combination treatment of cisplatin and anti-CD133 CAR-T cells effectively inhibits tumor progression and reduces the infiltration of CD133-positive stem-like cells in three different xenograft models (180). Although the CRISPR/Cas9 technology has brought breakthrough progress in cancer immunotherapy, the off-target risks of its gene editing and long-term safety (such as potential genomic instability) still need long-term tracking and verification. The CAR-T therapy, although showing potential in hematological tumors, faces side effects such as poor penetration in solid tumors and cytokine storms, as well as practical bottlenecks including high costs of personalized preparation and limited patient adaptability. These issues urgently require technological breakthroughs to promote its wider application.

### 5.4 Clinical trial treatment

In clinical trials, the development of biomarkers is of great importance as they can dynamically monitor the CSCs load and guide personalized treatment. In recent years, researchers have successively developed a variety of biomarkers. For example, genes such as TBX15, IGF1, GATA2, PITX2, SNAI1, and VCAN have been discovered in mesenchymal stem cells from elderly donors, which can serve as potential biomarkers to diagnose the senescence state of donor mesenchymal stem cells and evaluate whether MSCs from elderly donors can be used for clinical treatment (181). Clinical improvement was observed at a median follow-up of 180 days after MSCs transplantation, including a decrease in the SLE Disease Activity Index score, urine protein/creatinine ratio, and erythrocyte sedimentation rate, as well as an increase in the levels of complement C3 and C4, hemoglobin, and platelets. Metabolomic results showed a 35% increase in the level of thiamine monophosphate (TMP), confirming that TMP is a potential biomarker that can predict the efficacy of MSCT in the treatment of SLE (182).

However, clinical trials have shown that when MSCs are infused via the traditional intravenous (IV) route, due to the first-pass effect of the lungs, most of the cells are trapped, resulting in a significant reduction in the number of cells reaching the target organ (183, 184). Local injection of MSCs into the renal artery is significantly more effective than intravenous injection in reducing renal fibrosis induced by ischemia-reperfusion injury (IRI). MSCs injected via the artery survive in the injured kidney for more than 21 days, while those injected intravenously survive for less than 7 days. This difference is related to the targeting of the renal tubular injury area caused by IRI, suggesting that renal artery injection of MSCs may be an effective strategy to prevent the progression of acute kidney injury (AKI) to chronic kidney disease (CKD) (185). Similarly, in a streptozotocin (STZ)-induced diabetic mouse model, the method of intra-arterial (IA) injection of bone marrow-derived MSCs (BM-MSCs) directly targeting the pancreas significantly improves the hyperglycemic response compared with intravenous injection. Mechanistically, MSCs injected intravenously have low pancreatic colonization efficiency due to entrapment in the pulmonary circulation, while IA delivery can avoid the first-pass effect and ensure that more cells migrate to the injured site to exert therapeutic effects (186). It should be noted that although arterial injection of MSCs avoids pulmonary entrapment, the complexity of the operation limits its possibility of generalized application of the treatment.

## 6 Conclusion

Osteosarcoma remains a formidable challenge in oncology, with CSCs serving as the linchpin of therapy resistance, metastasis, and recurrence. This review synthesizes critical advances in understanding the dual roles of stem cells in OS pathogenesis and therapy: 1.CSC-driven mechanisms involving dysregulated Wnt/β-catenin, Notch, and Hedgehog pathways (e.g., CD133/CXCR4-mediated stemness) underpin chemoresistance and immunosuppression. 2.MSC paradox: While engineered MSCs offer promise as tumor-homing drug carriers (e.g., IDD-1040-paclitaxel complexes) and immunomodulators, their capacity to differentiate into cancer-associated fibroblasts (CAFs) via TGF-β secretion exacerbates tumor progression. 3.TME dynamics: Hypoxia-induced HIF-1α activation and M2 macrophage polarization create a permissive niche for CSC resilience, necessitating microenvironment-targeted strategies.

Emerging therapies—including CRISPR-edited CD133-directed CAR-T cells, CSC-specific epigenetic inhibitors (AZD1080, DNMTi/HDACi), and intra-arterial MSC delivery—show preclinical efficacy but face translational barriers: 1. CSC heterogeneity and plasticity compromise the durability of targeted therapies. 2. MSC batch variations, tumor-promoting risks (e.g., fusion-mediated PD-L1 upregulation), and pulmonary entrapment impede delivery efficiency. 3. Antigen loss associated with CAR-T therapy and the off-target effects inherent in gene editing require solutions.

Future efforts must prioritize: 1. Biomarker-guided combinatorial regimens (e.g., CSC pathway inhibitors + immune checkpoint blockers) to overcome compensatory activation. 2. Advanced delivery platforms: Microenvironment-responsive MSC engineering and optimized routes (e.g., intra-arterial injection) to enhance tumor localization. 3.Translational integration: Multi-omics profiling of CSC subpopulations, patient-derived organoid models, and AI-driven drug screening to accelerate clinical validation.

Ultimately, a paradigm shift toward stem cell-focused personalized therapy—addressing CSC eradication while harnessing MSC delivery precision—holds transformative potential for OS treatment.
